# Microfluidic Approaches for Manipulating, Imaging, and Screening *C. elegans*

**DOI:** 10.3390/mi7070123

**Published:** 2016-07-19

**Authors:** Bhagwati P. Gupta, Pouya Rezai

**Affiliations:** 1 Department of Biology, McMaster University, Hamilton, ON L8S 4K1, Canada; 2 Department of Mechanical Engineering, York University, Toronto, ON M3J 1P3, Canada; prezai@yorku.ca

**Keywords:** *C. elegans*, microfluidics, live imaging, electrotaxis, neurobiology, high-throughput screening, drug discovery

## Abstract

The nematode *C. elegans* (worm) is a small invertebrate animal widely used in studies related to fundamental biological processes, disease modelling, and drug discovery. Due to their small size and transparent body, these worms are highly suitable for experimental manipulations. In recent years several microfluidic devices and platforms have been developed to accelerate worm handling, phenotypic studies and screens. Here we review major tools and briefly discuss their usage in *C. elegans* research.

## 1. Introduction

*C. elegans*, commonly referred to as the worm, is the leading animal model for biomedical research and screening of drugs and drug targets [1,2]. As a multicellular system, *C. elegans* offers many experimental advantages including small size, transparency, ease of culturing, rapid growth, large brood size, cell lineage, and a relatively compact genome (~100 Mb) that is fully sequenced. The animal consists of two sexes: hermaphrodites (that are essentially females but produce a limited number of sperm initially before switching to make oocytes) and males. Hermaphrodites produce progeny using their own sperm as well as from males following mating. Fertilized eggs hatch to become L1 larvae, which then transition through L2, L3, and L4 larval stages, each separated by molting, to become adults in less than three days at 20 °C. The nervous system of the adult worm consists of 302 neurons whose interconnections are fully mapped [3,4]. Research has shown that many of the cellular and molecular processes in worms are conserved across almost all eukaryotes [5,6], making it a highly relevant system to understand human biology, investigate the mechanisms of diseases, and carry out drug discovery experiments. However, the small size of *C. elegans* (approximately 1 mm length and 60 μm width) and its continuous undulatory locomotion imposes a significant challenge in manipulations that involves automated and high throughput methods. The research community has therefore been in continuous search for technologies/techniques to address this issue.

Miniature devices such as microfluidic chips and Micro-Electro-Mechanical Systems are increasingly being used in biological research and drug discovery. These devices offer many advantages such as the small size, low consumption of reagents, ability to manipulate small objects and automate operations, increased throughput, small footprints, and excellent safety and reliability. Microfluidics in particular deals with the study and control of fluids and nano- to micro-scale objects inside miniaturized environments via incorporation of operation automation tools such as micro-pumps and micro-valves. Microfluidic tools have been used successfully to manipulate biological fluids, cells, tissues, and even whole organisms [7,8]. In this review, we specifically focus on the applications of microfluidics in research involving *C. elegans*.

The usage of microfluidic chips for *C. elegans* experiments has grown rapidly in the last decade. The material commonly used in the fabrication of these devices is polydimethylsiloxane (PDMS), a polymer that is transparent, flexible, gas permeable, and is not toxic to worms. PDMS parts could be joined or bonded with other components made of glass, silicon, and steel to create complex devices. These devices can handle almost every step of worm manipulation and analysis including culturing, different treatments, and continuous monitoring of cellular and behavioral phenotypes. The existing *C. elegans* microfluidic systems could be classified into roughly three categories based upon their use: (1) devices to assist with routine laboratory procedures (e.g., culturing worms); (2) devices to perform particular types of experiments (e.g., monitoring neuronal activities); and (3) devices for high-throughput screens. This review summarizes developments in these categories, highlighting unique features of different devices and their advantages in accelerating research.

## 2. Microfluidic Devices to Assist with Routine Procedures

Many routine experiments in *C. elegans* labs such as phenotypic observations require collecting worms of a certain age or phenotype, and culturing them for a set duration before imaging. Routine protocols to process animals for these purposes are tedious due to their manual nature, which is labor-intensive, time-consuming, and does not readily scale up. For example, collecting animals of a specific developmental stage from a culture of mixed stages requires picking them individually which could take hours. Depending on the operator, progress and quality of output could vary significantly. Microfluidic approaches can overcome many of these limitations by automating common steps, leading to increased throughput, higher accuracy, reproducibility, and lower cost of operations. Some of the most common procedures are discussed below.

### 2.1. Worm Sorting

One of the most common needs in *C. elegans* labs is sufficient quantities of animals of a certain type, e.g., developmental stage, size, sex, or phenotype. In late 1990s, Union Biometrica (http://www.unionbio.com) developed an automated worm sorting platform, termed COPAS (Complex Object Parametric Analyzer and Sorter), for automated sorting of worms based on their size and certain other features. Subsequently an advanced sorting platform, termed ”BioSorter”, was released that offers a rich set of features and modularity. Although both these non-microfluidic platforms are useful in *C. elegans* studies, they are quite sophisticated in terms of operation and maintenance. Furthermore, their cost is prohibitive for the majority of laboratories. As an alternative, several microfluidic sorting devices have been fabricated in the last decade that are affordable and easy to operate. Table 1 provides an overview of these techniques and their main features.

**Figure 1:**
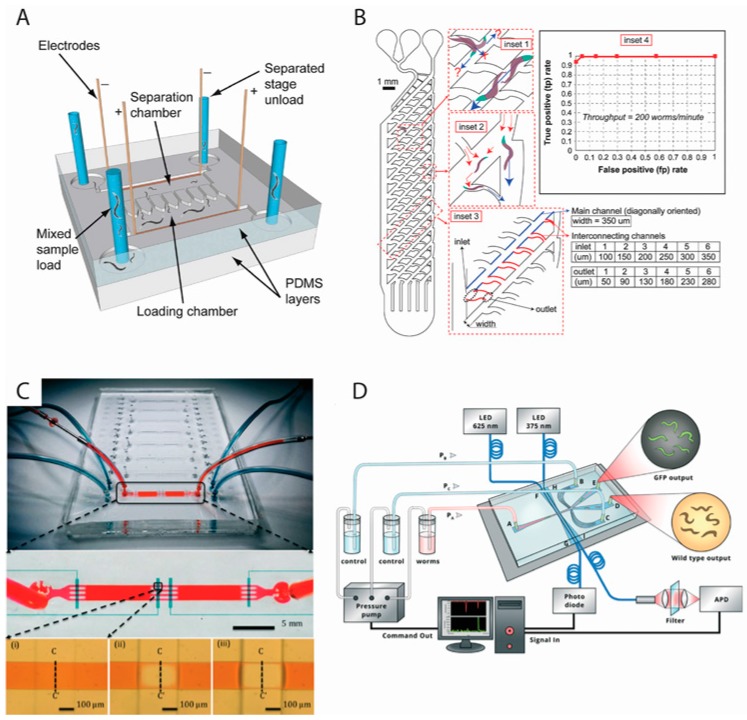
Microfluidic devices for *C. elegans* sorting using (**A**) electrotaxis (Rezai et al. [10], (**B**) mechanical microstructures (Solvas et al. [12], (**C**) deflectable membranes (Dong et al. [17], and (**D**) fiber-based fluorescent detection (Yan et al. [15]). Reproduced with permission from The Royal Society of Chemistry. Panel (**A**) shows the electric trap-based sorting device. Loading chamber contains mixed stage worms. Sorted worms accumulate in the separation chamber and are recovered via unload channels. The smart maze concept is shown in panel (**B**). The four insets show worm orientation (inset **1**); flushing of small larvae (inset **2**); dimensions of the successful design (inset **3**); and successful recovery of adults in an experiment (inset **4**). The deflectable membrane device in panel (**C**) shows eight individual worm selection units (one of these connected with tubes). The fluidic and valve control channels are enlarged to show details. The fluidic path is squeezed upon activation of the control valve. The device in panel (**D**) contains inlets and outlets for worms and buffer. The optical fiber channels (LED 625 and 375 nm) are used to differentiate between wild-type and fluorescing worms. Refer to respective references for more details.

Our group was the first to demonstrate worm sorting based on the electrotaxis speed of the animals [9,10]. Electrotaxis is the tendency of worms to respond inherently to desirable electric signals and move towards the favorable electrode (negative electrode in case of *C. elegans*). Using a device (Figure 1A) that consisted of a worm loading and a collection chamber interconnected with a series of parallel thin microchannels that acted as local electric field traps, we succeeded in obtaining synchronized cultures of L3, L4, and adult animals from populations of certain mixed stages (e.g., L3 mixed with L4). The mixed population of worms was passed slowly across the loading chamber while being exposed to a perpendicular electric field that induced them to move laterally towards the narrow traps. However, since the electric field in the traps was unfavorable to older (larger) animals, they ended up restraining from entering the trap while the younger (smaller) worms conveniently passed through the channel and became separated from the mix. The device could reach a throughput of 78 worms per minute and also separate neuron- or muscle-defective worms from normal worms.

Subsequently, other labs also reported electrotaxis-based microfluidic sorters. Maniere et al. [11] constructed a “worm electrophoresis” unit that is essentially a gel box containing a 10 cm long agar track filled with buffer. Worms were placed at one end and allowed to swim under the influence of the DC electric field. The animals were spatially sorted along the runway based on their speed. Although the device was slow to operate (up to an hour for each run to complete), it could distinguish movement-defective worms from wild type as well as separate older animals from younger ones. Han et al. [13] fabricated a device containing multiple parallel micro sinusoidal channels. The channel was designed such that they presented physical barriers and required worms to make efforts as they moved in the presence of an electric field. Using their setup, authors succeeded in sorting worms from a mixed culture of all stages and reported a throughput of roughly 250 adults an hour with 95% accuracy. An earlier investigation [9] showed that electric field does not severely affect the viability of worms, thus supporting the use of electrotaxis approach in long-term post-sorting assays.

While the above microdevices are useful, they lack the ability to sort all developmental stages simultaneously. To this end, a PDMS-agarose hybrid device was developed by Wang et al. [16] that relied on the sensitivity of *C. elegans* to electric field strength. It was earlier shown that worms, when exposed to the electric field on an open gel surface, prefer to move towards the cathode at an angle rather than in a straight line [18]. Wang et al. [16] found that this angular movement (termed “deflecting electrotaxis”) varies for different stages of worms even at a constant electric field. The older worms tend to deflect more than younger stages. Based on the observation of deflecting electrotaxis, they designed a fan-shaped structure, from −50° to +50°, consisting of channels at 5° intervals that originate from a central worm loading spot. Mixed stages of L2, L3, L4, and adult worms were successfully sorted using this setup at a throughput of ~56 worms per minute. The device was also able to sort worms of different sizes as well as separate males from hermaphrodites.

Besides electrotaxis, researchers have developed microfluidic devices that utilize mechanical methods to sort animals. Four reports [12,14,17,19] have described the use of channels, pillars, variable size filters, and thermosensitive hydrogels to achieve a similar goal. Solvas et al. [12] developed a device (Figure 1B), termed “smart mazes,” that consisted of a main channel containing a variety of micro-features such as pillar arrays, pools, and “smart” filter and mazes of different dimensions to passively sort worms based on their size. Up to 94% accuracy was observed in sorting adults from larvae. Typically 200–300 worms were processed per minute, which could go up to 1200 worms per minute under some conditions. Although the device is limited in applications, e.g., due to fewer stages being sorted, and the lack of mutant sorting, it could still be useful in many routine assays. Another study by Ai et al. [14] incorporated micro-pillar structures in their devices to sort worms using a flow filtration approach. Each device contained geometrically optimized arrays of pillars such that the spacing was suitable for each developmental stage. While each of these devices could be operated alone to separate two populations that were mixed together, when connected in a serial fashion, they allowed efficient sorting of worms containing a mixture of L1–L4 larvae and adults. The throughput ranged roughly between 130 and 180 worms per minute, which is suitable for most routine biological assays. Investigations of physiological processes (pharyngeal pumping rate and body bend frequency) and three-day survival in L4-stage worms sorted in the device showed that the sorting experiments had no detrimental effect on worms’ physiology and fecundity. Finally, earlier this year, Dong et al. [17] reported a sorting device that was based on the principle of adjustable filter to allow passage of worms of certain diameter. The device (Figure 1C) consisted of a straight microchannel with one inlet and one outlet, two worm collection chambers, and four deflectable membrane valves that act as adjustable filter. The filter parameters were altered using pressure applied onto the membrane through a syringe pump. Mixtures of worms of two adjacent developmental stages, e.g., L3 and L4, could be efficiently and rapidly (roughly 200 worms per minute) separated. Although authors suggest that multiple stages of mixed cultures can also be sorted, it was not demonstrated in their study. Another feature of this setup was the separation of embryos from adults, which can be useful in many studies.

The approaches discussed so far deal with isolating synchronized animals by relying on differences in their sizes and movement responses. Since many *C. elegans* experiments require transgenic animals that express Green Fluorescent Protein (GFP) reporters, it would be highly desirable to perform sorting based on their fluorescence. A number of automated systems for fluorescent-based detection and sorting in microfluidic devices have been reported [15,20,21]. The device by Yan et al. [15] (Figure 1D) can sort fluorescing animals using optical fiber detection and laminar flow switching. The system used two pairs of closely placed fibers, one for sensing the presence of nematodes as they pass through the light path and the other for detecting fluorescence. Sorting then occurred into the arms of a downstream Y-shaped channel that was controlled by flow pressure that guided worms to a desired arm based on their fluorescence status. The entire operation was controlled by customized software and did not require a microscope. This platform could sort worms at an optimum speed of 700 worms per hour with almost 100% accuracy.

In summary, the passive sorting methods that involve mechanical obstacles in microfluidic channels allow separation of animals based on their size at reasonably high throughputs. However, they are unable to perform sorting using other characteristics such as marker gene expression or behavioral responses. In these cases active methods such as electrotactic or fluorescence-based sorting are more desirable.

### 2.2. Worm Culturing

Many of the in vivo studies of biological processes in *C. elegans* require maintaining cultures for an extended period of time. Worms need to be monitored and periodically examined for changes in cellular processes. Microfluidic devices have been fabricated to streamline culturing and observations of worms [22,23,24,25,26,27,28,29,30,31]. In one of the earliest studies by Kim et al. [32], authors reported a compact disc (CD)-shaped device to culture worms for several days. This simple tool relied on the centrifugal force to drive the food diagonally in a rotating CD from inner nutrient reservoirs into cultivation chambers to feed the worms and to eject the waste from these chambers to outer waste reservoirs. For observation purposes, a small aliquot of the culture was removed periodically and examined under the microscope. The device was able to grow animals for up to three generations (in two weeks) without affecting their growth and behavior in any obvious way. However, due to its simple design, it could not distinguish between progeny and parents.

**Figure 2:**
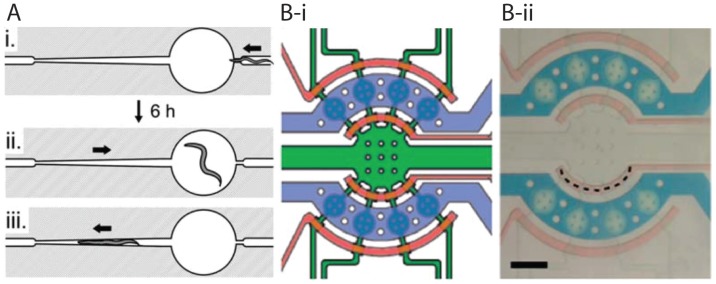
Microfluidic devices for culturing and long-term studies of worms inside cultivation chambers while immobilization and imaging is performed by (**A**) tapered microchannels (Hulme et al. [23] or (**B**) responsive hydrogels (Krajniak et al. [29]. Reproduced with permission from The Royal Society of Chemistry. The tapered microchannels connected to growth chambers (panel (**A**)) allow single worms (early L4 stage) to enter into each chamber. Arrows indicate the direction of liquid flow. Once the worm has grown it is unable to escape the chamber. For imaging purposes, the worm is temporarily immobilized in the tapered region. Panel (**B**) The two sub-panels **B-i** and **B-ii** show the device that contains valves (**red**) to control fluid flow, channel for flowing heating liquid (**light** **blue**), and eight worm culturing chambers (two sets of four) and a central waste outlet tube connected to a loading channel (**green**). Refer to respective references for more details.

While useful, the compact disc system has some limitations. For example, it lacks the ability to track individual animals and to perform automated on-chip imaging. To address this, Hulme et al. [23] developed a new device. The device (Figure 2A) consists of chambers for long-term culturing and locomotion studies, connected to side narrow microchannels used for immobilization, imaging, and body size measurements at different time slots. While the device could not monitor early larval stages, it was capable of lifelong culturing of single worms from L4 stage onwards by trapping them individually in chambers. Up to 16 adults could be cultured at a time in parallel chambers, which accelerated studies of age-related behavioral and physiological changes. The authors successfully reported that there is a close correlation between the end of growth day and decline in swimming frequency with the lifespan of worm.

In addition to cultivating the worms in chambers and moving them to immobilization channels for imaging, researchers have also used the concept of droplets and responsive reversible gels to perform long-term investigations [19,29,30,31]. For instance, Krajniak and Lu [29] developed an integrated microfluidic device (Figure 2B), that consisted of an array of eight microchambers controlled by surrounding channels and valves, and successfully showed culturing, immobilization and imaging of animals on a single platform. Animals were loaded from a single inlet into eight culturing chambers. For imaging, Pluronic F127 (PF127), an amphiphilic block copolymer (PEO99-PPO67-PEO99, PEO: Poly(ethylene oxide) and PPO: poly(propylene oxide)), was injected into the chambers from the same inlet and gelled by heating. This polymer is highly viscous at low temperatures (e.g., 15 °C) but acquires gel-like properties upon raising the temperature (e.g., 21 °C), making it possible to reversibly immobilize animals. Since PF127 does not exhibit autofluorescence and has no effect on the viability and development of worms, animals could be successfully monitored from the L1 stage to adulthood. The gel-based immobilization technique was also utilized in a recent publication by Cornaglia et al. [17] to monitor in vivo protein aggregation in *C. elegans* models of two neurodegenerative diseases, namely amyotrophic lateral sclerosis (ALS) and Huntington’s disease (HD). The authors demonstrated the progression of mutated human superoxide dismutase 1 (SOD-1) tagged with Yellow Fluorescent Protein (SOD1-YFP) into the body wall muscles of individual worms over several days. They also precisely localized fluorescent proteins in tissues, and monitored the time-lapse and sub-cellular evolution of single aggregates over time.

Based on the above discussions, it is clear that the selection of an appropriate microfluidics method for culturing *C. elegans* will depend on the unit operations to be performed for monitoring processes and the length of assay. For instance, if there are multiple chemical exchanges desired in the assay or if the experiments involve offspring, then the chamber-based methods that incorporate nutrient and waste exchange are better choices. However, if studies require observing animals for a short duration at a lower throughput then hydrogel immobilization approach may be more desirable.

### 2.3. Worm Immobilization Methods for Developmental, Morphological, and Physiological Studies

Because of their microscopic size and continuous movement, worms need to be immobilized for routine observations of live processes. For developmental studies, imaging is often performed at multiple time points throughout the life of animal, so it is important that immobilization is reversible and does not cause harm. This is typically achieved by the use of agents such as anesthetics and glue. However, both these approaches are less than satisfactory due to their potential toxicity and slow pace. Also, the use of glue precludes monitoring the same animal at multiple time points. Alternative approaches such as instant cooling, compression, thermosensitive hydrogels, and exposure to gases have been successfully attempted.

Krajniak et al. [29] used the temperature-sensitive PF127 gel (mentioned in Section 2.2) to immobilize animals for developmental studies. Up to eight worms could be studied simultaneously in their device (Figure 2B), each maintained in individual chambers and supplied with food. For imaging purposes, a solution of PF127 was flooded in culture chambers. Temperature increase was achieved by circulating hot water via separate channels passing above the worm cultivation channel, shown in Figure 2B. Although the platform makes it possible to perform time-lapse studies at a high speed, the addition of temperature control modules makes the system somewhat complex.

**Figure 3:**
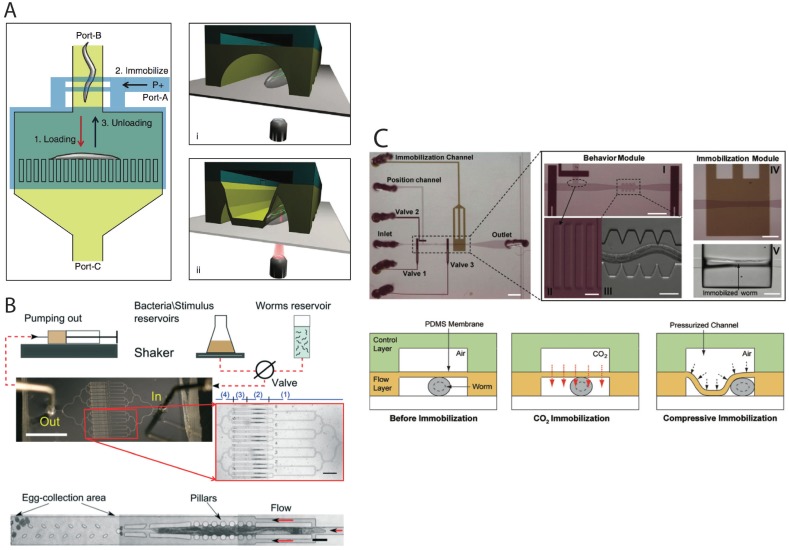
Microfluidic devices to immobilize *C. elegans* using (**A**) deflectable membrane (Gilleland et al. [33]; (**B**) tapered microchannels (Kopito and Levine [34]); or (**C**) CO_2_ exposure (Chokshi et al. [35]. Reproduced with permissions from The Royal Society of Chemistry and Macmillan Publishers Ltd. Nature Protocols. Panel (**A**) shows the chip containing an array of narrow channels to apply suction pressure. Worm is loaded/removed through port-B and restrained by the narrow channel array. Pressure through port-A causes the compression layer to move downwards and immobilize the worm (explained on the right). Releasing the pressure allows the worm to be recovered. The WormSpa device, in panel (**B**), contains four regions for worm loading and distribution (**1**), egg chambers (**2**), egg collection (**3**), and outflow (**4**). The device for CO_2_ based immobilization is shown in panel (**C**). It contains modules for behaviour assay (first row of pictures) and immobilization (second row of pictures). Refer to respective references for more details.

Another attractive approach to image worms in a microfluidic setup is to physically trap them in a narrow space. Gilleland et al. [33] have described a detailed protocol for setting up a pressure-based immobilization imaging platform that uses a flexible PDMS membrane to restrain the motion of worms in the channel while they are being imaged (Figure 3A). The device consisted of two layers of PDMS microchannels bonded together with the flexible membrane sandwiched in between. The bottom layer allows for loading and unloading the worms into the immobilization section of the device. The top layer is used to apply a pneumatic pressure on the membrane in order to deflect it down onto the loaded worm. Animals can be recovered from the device upon removing pneumatic pressure from the flexible membrane. Subsequent studies showed no visible sign of stress or damage in such worms following recovery.

An alternative mechanical method to keep worms steady at one location is to use tapered microchannels, as shown in Figure 2A. This method does not require a moving part in the device; instead the worm is gently pushed longitudinally to a narrow region of the channel such that it has no room to move any further. Such a confinement approach is desired in scenarios where animals are exposed to chemicals in a controlled manner or there is a need to collect embryos. For instance, Kopito and Levine [34] used the principle of tapered channels in their device that held animals in parallel units, each consisting of two rows of micro-pillar sidewalls instead of solid walls (Figure 3B). The pillars allowed worms to wiggle slightly without compressing while making it easier for embryos to escape by frequent washes. Their chips, termed “WormSpa,” could be used to simultaneously culture up to 64 adults in the long term while monitoring physiological changes and gene expression under a microscope. Our group has also developed a simple micro-structured device to instantaneously confine several animals on a regular glass slide in order to image them for up to several hours [36]. These confinement approaches do not appear to affect the growth and reproduction of the animals, making them suitable for many *C. elegans* applications.

Two papers [35,37] have compared the pressure-based approach with CO_2_ exposure through a thin membrane as an anesthetic gas to immobilize animals in microfluidic devices (Figure 3C). Chokshi et al. [35] showed that 1–2 h long exposure of CO_2_ caused no apparent harm to animals and they recovered successfully. However, longer exposures (3 h and beyond) resulted in lethality. The pressure-based immobilization, using a deflectable PDMS membrane, implemented in both these studies allowed short term (minutes) immobilization and imaging without affecting the recovery. While both approaches are useful, CO_2_ is reported to have a detrimental effect on neuronal processes [37], thereby limiting its applicability.

An interesting method to immobilize animals by laser heating was recently described by Chuang et al. [38]. The authors developed a new device to rapidly immobilize worms for routine applications such as morphological characterizations, in vivo study of cellular processes using fluorescent reporter, and targeted cell ablations. The unique feature of the device is that it uses a laser beam combined with electric field to heat the liquid medium to 31 °C causing paralysis of animals in less than 10 s, a technique termed “addressable light-induced heat knockdown (ALINK)”. While useful in some applications, this approach is not well suited for studies requiring repeated immobilizations because of high lethality in subsequent heat treatment cycles. Moreover, individual worms cannot be easily and reliably recovered for follow-up observations.

The above approaches have focused exclusively on larvae and adults. However, microfluidic techniques have also been successfully applied to manipulate embryos, allowing for automated and high throughput arraying of many embryos at predetermined locations on a chip, physiological maintenance over time, and long-term live imaging to study embryogenesis. Cornaglia et al. [39] recently developed a new device to isolate and image embryos with high accuracy. Their device contained two chambers, one for culturing worms and the other for incubating embryos. Up to 20 embryos could be cultured simultaneously, each resting in stable positions in little incubators. The embryos were obtained from adult worms that were maintained in a separate culture chamber. Embryos could be imaged over time, which was useful for morphological and gene expression studies. Gene expression and developmental events were captured using a fully automated multi-dimensional imaging system.

All in all, the immobilization methods summarized in this section are suitable for a wide range of applications. The choice of a specific approach in an experiment will depend on the immobilization duration and the readout signal desired. For instance, CO_2_ exposure can be used to immobilize animals for up to 2 h, but if neuronal processes or pharyngeal movements are to be investigated then it is better to avoid anaesthetics. For experiments requiring manipulations such as neuronal ablation, one could utilize mechanical, chemical, and even temperature-based approaches to quickly restrain worms for short durations.

### 2.4. Microinjection

Among routine procedures in *C. elegans* laboratories is the injection of DNA, proteins, and chemicals for the purpose of examining biological processes. When DNA pieces are injected into the germline, they may be incorporated in the genome resulting in the generation of transgenic animals. Traditional microinjection protocol involves placing animals in a particular orientation on a glass coverslip containing a thin layer of dried agarose and immersing them in oil to slow down desiccation. This step is rather difficult because it requires that animals have sufficiently adhered to the slide and do not wiggle too much. Next, they are placed on a microscope and an injection needle, filled with the desired solution, is carefully brought into the field of view and manually inserted in the worm to inject the contents. Finally, worms are recovered from the slide. Due to the slow and labor-intensive nature of this procedure, it can take hours even for an experienced person to inject a sufficient number of worms for a single experiment.

**Figure 4:**
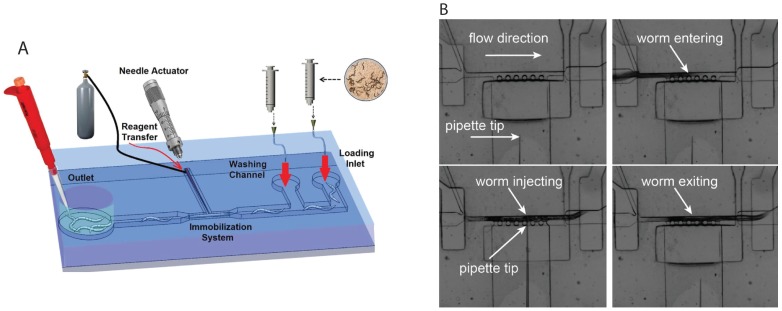
Microfluidic devices for microinjection in (**A**) closed microchannels (Ghaemi [41] and (**B**) open chambers (Song et al. [43]. Panel (**B**) reproduced with permission from American Institute of Physics Publishing. The device in panel (**A**) contains worm loading and washing channels (on the right) and an outlet for collecting injected worms. Worm is immobilized in the middle region for injection. The image frames in panel (**B**) show a sequence of worm loading, injection, and flushing. Refer to respective references for more details.

In recent years, attempts have been made to develop microfluidic devices to automate this protocol. In 2013, we reported such a device (Figure 4A) [40,41]. This “T” shape unit was fabricated by pre-loading the injection needle into the T-channel before bonding the device layers together, hence ensuring that the needle tip is aligned properly with the narrow worm trapping channel. Injections were performed by confining worms in the narrow trap using suction pressure. The movement of the needle and injection were achieved by applying 200 kPa pressure pulses with 1 s duration using an attached micromanipulator. Success with the generation of transgenic animals was also demonstrated [41]. The process of introducing worms into the device and recovery is still performed manually, but could be automated in the future. Another device by Zhao et al. [42] used a suction-based approach to immobilize animals while they were being injected. The injection step was controlled by hand. Earlier this year, Song et al. [43] reported an automated system capable of injecting 6–7 worms per minute with a success rate of 77.5%. The device (Figure 4B) consisted of microvalve-controlled loading and unloading channels connected to a narrow microchannel to house the worm, a set of perpendicular side suction channels for immobilizing animals in the narrow channel, and a 5 μm-tip micropipette controlled automatically by a three degree of freedom micromanipulator for precise injection of chemicals into worms. The throughput of this device was increased by incorporating a robotic system to automate the operation, real-time image processing to operate the needle, and the process of injection. Overall, future application of the microfluidic approach to microinjection is promising.

## 3. Microfluidic Devices to Assist with Specialized Assays

In addition to the routine procedures of handling *C. elegans*, as described in the previous section, a large number of microfluidic tools have been developed to facilitate research in this animal model. These include investigations of specific processes such as movement, cell morphology, gene expression, learning and memory, toxicology, and aging. Key applications are summarized below.

### 3.1. Study of Movement Responses in Microstructured Environments

The natural habitat of *C. elegans* is complex and requires animals to probe the environment in order to live, feed and reproduce. Mechanosensation, i.e., the response to touch, plays a vital role in these processes. Microfluidic devices have been designed to investigate the mechanosensory response of animals in the laboratory. One of the earlier devices, reported by Park et al. [44] was constructed of agar and not PDMS. It contained 300 μm diameter circular posts in a 200 mm × 20 mm × 0.11 mm chamber arranged in a square grid configuration. The device was placed in a petri dish with the topside open and accessible. The chambers were filled with buffer prior to placing worms inside them. Examination of movement responses of different worms revealed that animals rely on touch to move efficiently in such an environment since mechanosensory mutants *mec-*4 and *mec-*10 were slower than the wild type. Increasing the post spacing from 400 to 475 μm resulted in higher swimming speed but any further increase in the spacing had an opposite effect. The device could be used to study the movement of worms, and possibly screen for new mechanosensory and uncoordinated mutants.

Another device, reported by Parashar et al. [45] contained micro-sinusoidal shaped structures to examine movement responses of worms. Channels of different shapes (ascending and descending in amplitudes from 135 to 399 μm and from 135 to 10 μm, respectively) were tested, and it was observed that animals moved faster in certain configurations but slower in others. The speed was the highest (average of approximately 300 μm/s) in a channel with sinusoidal shape amplitude of around 200 μm. Analysis of movement defective strains (*lev-*8 and *unc-*38) revealed an almost 50% reduction in velocities of worms in these channels, suggesting that such a setup may be useful in screening animals for locomotion defects.

Subsequently, two devices were reported that contained micro-pillar structures for measuring the forces exerted by worms on their microenvironment [46,47]. Johari et al. [46] used PDMS pillar elastic deflection concept to measure the force, and found that despite changes in the placements of pillars in a “honeycomb” or “lattice” configuration, worms continued to move in a sinusoidal pattern but their locomotion speed, undulation frequency, and body force changed significantly. Measurement of forces applied on pillars revealed that the mid-body region generates the maximum force. The device by Qiu et al. [47] was designed to expose worms to a particular frequency of light that they tend to avoid and measure force generated during forward or backward movements, or omega turns in the structured environment. A LabVIEW program (National Instruments, Austin, TX, USA) was also developed to assist with image capture and data analysis. The platform can be used to examine the locomotive behavior of animals and correlate it with neuronal function.

### 3.2. Study of Cellular Processes

One of the biggest advantages of *C. elegans* as a model organism is its transparency, which enables real-time in vivo study of cellular events. There is no need to sacrifice animals for observing cells and tissues, performing surgical manipulations, and examining gene expression using fluorescent (GFP) reporters. In fact, the worm model allows these experiments to be performed in live condition and longitudinal manner. Over the years, several groups have reported microfluidic devices to automate, streamline, and accelerate such assays.

Studies of neuronal activities require keeping animals stationary for several hours during imaging processes. It is critical that animals are not paralyzed, which precludes the use of anesthetics, and that they are not subjected to stress during the period of observation. To achieve this goal, Mondal et al. [37] fabricated a device consisting of a pressure-deflecting PDMS membrane to immobilize animals. The device permitted long-term imaging of neuronal transport processes (up to an hour) with high resolution without causing harm to neurons, something that is not possible with the traditional anesthetic-based immobilization methods. The authors demonstrated the capability of their device to investigate subcellular events such as synaptic vesicle transport, Q neuroblast divisions, and mitochondrial transport in early larval stages, which was not observable in worms of the same age that were anesthetized with chemicals like levamisole. In addition to worms, authors also used the device to successfully study processes in *Drosophila* larvae. However, this setup has very low throughput and is only suited for detailed cell biological studies in a small set of animals.

Another way to restrain animals is to trap just part of the animal body. This approach was recently demonstrated by Hwang et al. [48] in a series of optogenetic experiments. In such a system, worms were illuminated with a specific wavelength of light that causes an escape response and observed while trapping them at both ends using two pneumatically-controlled microvalves. To accelerate data collection the device allows simultaneous image captures from 16 parallel channels. The authors used this setup to examine the activities of body wall muscles. They successfully studied the roles of 15 genes that encode sarcomere proteins in striated body wall muscles and showed that these genes are required in various steps of muscle function. Several key parameters of muscle kinetics and rate constants of contractions and relaxations were determined. The findings led to an improved understanding of how muscles control locomotion in worms.

In addition to imaging, the pressure-based immobilization technique can also be useful in other procedures such as laser-assisted surgeries. In one of the earliest reports [49], the authors described a device to perform long-term observations of neuronal processes with or without laser-assisted ablation of synapses. The device contained several parallel channels that were large enough at one end to allow easy uploading of worms using a syringe pump-driven fluid flow. The channels were tapered to immobilize animals as they move down the channels. Time-lapse imaging was performed using a CCD camera. To ablate synapses, short pulses of a UV laser were used. The setup was successfully used to monitor neuronal processes for up to 4 h. Another surgical approach, developed in recent years, involved the use of a genetic system. Lee et al. [50] used a KillerRed (KR) system to perform rapid neuronal ablation in a device and image behavior of animals for up to 24 h. The KillerRed protein is a genetically-encoded photosensitizer that is activated upon exposure to green light of the wavelength range 540–590 nm. Activation of KR in a cell causes production of reactive oxygen species, resulting in its death [51].

While the above approaches of imaging immobilized individual animals offer a detailed view of cells and cellular processes, they lack the ability to capture dynamic changes in real time as animals are interacting with the environment. Two papers [31,52] have reported new systems to address this limitation. Larsch et al. [52] developed a microfluidic arena of 3.28 × 3.28 mm^2^ and 50 μm depth that could be monitored using a high numerical aperture objective (2.5×/0.12 N.A. or 5×/0.25 N.A.) and sensitive low-noise CCD camera. This device could track up to 20 worms in real time and allow measurements of neuronal activities using GFP-based genetically encoded calcium indicators [53]. The setup enabled the authors to expose animals to different odors and monitor neuronal activities following changes in their movement responses. Overall, the approach is powerful because it allows for mapping of neuronal signals while the organism is responding to external signals and making decisions. For example, the results revealed that odor-induced Ca^2+^ signals in the AWA chemosensory neuron were variable among animals, possibly due to differences in their development, epigenetic modifications, or other processes. Measuring such signals can help explain the mechanistic basis of behavior in animals as they encounter stimuli in the environment. In the other system [31], animals of specific developmental stages were introduced in a culture chamber and maintained by supplying with food and buffer exchange. For imaging purposes, a PF127 sol-gel polymer was utilized. As mentioned earlier in this review (Section 2.2), the viscosity of this polymer changes with temperature. When the chip temperature is raised to 25 °C using a thermoelectric module, it triggers the gelation of PF127, causing worms to immobilize and neuronal processes to be examined in detail. Two different human disease models (ALS and HD) were successfully studied using this platform, which revealed changes in protein aggregation in cells over time.

**Figure 5:**
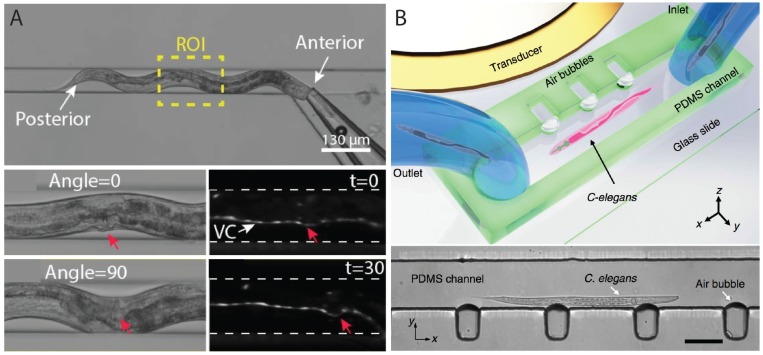
Microfluidic devices for multidirectional orientation and imaging of *C. elegans* using (**A**) rotatable glass capillaries (Ardeshiri et al. [54] and (**B**) acoustofluidic rotational manipulation (ARM) (Ahmed et al. [55]. Panel (**B**) reproduced with permission from Adapted by permission from Macmillan Publishers Ltd. Nature Communications. Panel (**A**) shows an adult worm inside the channel with the region of interest (ROI) in the middle. The worm is held by the negative pressure in the glass capillary. The two sets of brightfield and fluorescent images below show pre- and post-rotated views of specific neuronal processes (VC). Schematic view of the ARM device (**B**). It contains a piezoelectric transducer to generate acoustic waves. Air bubbles within sidewall cavities cause worms to rotate. The image below shows a mid-L4 worm trapped by oscillating bubbles. Refer to respective references for device details.

The three-dimensional shape of worms does not allow all cellular structures to be visible in any one given focal plane. Therefore, visualization of entire cellular structures requires either optical sectioning technique using expensive confocal systems or orienting worms in different directions while imaging. Ardeshiri et al. [54] were the first to demonstrate full rotation and multi-directional imaging of *C. elegans* in a microfluidic device (Figure 5A), using a rotary glass capillary that was used to pneumatically grab the worm from the anterior region and rotate it in a narrow microchannel. The authors successfully showed the 360° rotation of adult worms in the channel with on-demand immobilization and imaging of organs (vulva) and fluorescing neurons. Another study, reported earlier this year, came up with an elegant acoustic-based microfluidics approach to precisely and rapidly rotate worms and image them [55]. The device (Figure 5B) enabled acoustofluidic rotational manipulation (ARM) of *C. elegans* as well as single cells. During the process of worm loading, microbubbles were trapped into small microcavities created within sidewalls of the channel. This channel was placed in the vicinity of a piezoelectric transducer fabricated on a glass slide. The transducer generated acoustic waves that caused the microbubbles to initiate oscillatory motion resulting in steady microvortices, which rotated the animals that were in contact. By controlling the duration of waves (a few milliseconds), rotation angles could be controlled with high precision. For example, a worm could be fully rotated (360°) within 60 milliseconds with 5-millisecond pulses each causing 4° rotations.

Two drawbacks of the acoustofluidic technique are that the worms need to be anesthetized for imaging and that they cannot be accessed by external objects for further manipulation such as microinjection. The other approach involving micro-capillary to orient the worm [54] (Figure 5A) allows additional treatments, e.g., exposure to external stimuli, to be performed on animals.

### 3.3. Study of Chemotaxis, Electrotaxis, and Other Behaviors

As a metazoan, *C. elegans* responds to environmental stimuli or internal physiological changes by modifying its behavior. Because such responses depend on the functioning of the nervous system, assaying behavior provides valuable information about the state of neuronal signaling. Some of the commonly studied examples of *C. elegans* behavioral responses include chemotaxis (response to chemicals), thermotaxis (response to heat), phototaxis (response to light), and electrotaxis (response to electric field).

Traditional behavioral assays in *C. elegans* are carried out on agar-containing Petri plates. The procedures are slow to perform because they require a worker to carefully execute multiple steps including preparation of assay plates, handling worms, and data analysis. Furthermore, it is not possible to observe neurons in individual animals in such assays. In 2007 the Bargmann lab reported fabrication of two microfluidic chips to characterize neuronal and behavioral responses in worms at a high resolution [56,57]. One of these, the behavioral chip [56], had a single channel whose width was gradually decreased to 40 μm at one end (similar to the tapered channels discussed in Section 2.3). Worms were introduced into the channel one at a time and gently pushed towards the narrow end until they were trapped. Such animals could still generate a sinusoidal wave along the body length in an attempt to move, although they could not escape the small opening of the channel. Neuronal activities were measured in real time using GCaMP protein (genetically encoded Calcium indicator containing GFP, Calmodulin and M13 peptide sequences) that acts as a Ca^2+^ sensor. The chip allowed researchers to demonstrate that AVA neurons play important role in locomotion. The other chip, the olfactory chip [56,57], was used to trap worms in a narrow channel in such a way that their noses protruded into another connected channel where odors were delivered. This setup was used successfully to measure activities of several neurons including ASH and AWA to chemical stimuli.

**Figure 6:**
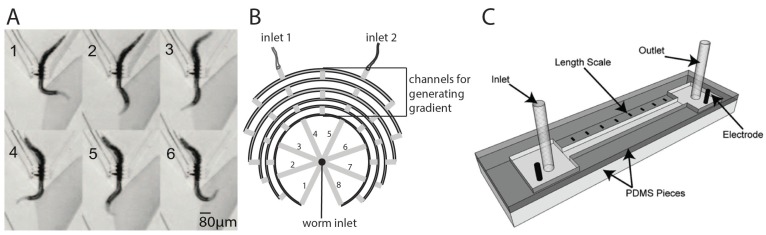
Microfluidic devices to investigate *C. elegans* behavior in response to (**A**) chemicals and heat (McCormick et al. [59]); (**B**) chemical gradients (schematic drawing of the device used by Hu et al. [60]); and (**C**) electric field (Rezai et al. [9]). Panel (**C**) reproduced with permissions from the Royal Society of Chemistry. Panel (**A**) shows head swinging of the worm in response to chemical exposure. In panel (**B**), the circular channel pattern used to generate the chemical gradient is shown. Worms enter into channels 1–8, which are 300 μm wide, 80 μm high, and 10 μm long depending upon their attractive responses to the NaCl gradient. The electrotaxis device in panel (**C**) contains electrodes to apply a DC electric field and a long channel for worm swimming. Refer to respective references for more details.

In recent years, several new microfluidic devices have been developed to further expand *C. elegans* studies. Albrecth and Bargmann [58] used microstructured areans consisting of hexagonally arranged cylindrical posts to examine odor-induced movement of worms. A custom automated tracking software was used to classify behavior into five distinct states: forward, pause, reverse, piroutette reverse (the reversal before an omega turn), and pirouette forward (forward motion after an omega turn). The responses were characterized, which revealed new quantitative and statistical insights into the odor-induced behavior of worms. This setup will enable future studies to understand neural circuits as animals encounter environmental stimuli and make decisions. McCormick et al. [59] reported a pair of microfluidic devices (Figure 6A), termed chemosensory and thermosensory devices, to measure the behavior of individual worms in response to chemical, thermal and osmotic stimuli. The unique feature of these devices was that responses were determined based on the head movement of semi-restrained animals. The lower half of such animals was fixed in the microfluidic channel while the upper half was free to perform side-to-side swings. Using these devices, one can reliably deliver chemical and thermal stimuli to animals and determine attractive and repulsive responses in the form of head swings. A new finding from this work was that both a rise and a fall in osmolarity lead to reversals in worms. The authors suggested that, with some modifications, it might be possible to perform neuronal imaging while recording the behavior of live and intact animals.

While the above setup enables single animal-based studies, chemotaxis assays are performed typically in batches consisting of hundreds of animals. A microfluidic device, developed by Hu et al. [60], simplifies such population-based experiments (Figure 6B). Repetitive flow splitting and mixing microchannels arranged in a tree-like design were used to generate linear gradients of sodium chloride (NaCl) across the device. Worms were loaded from the central outlet and their movement to chambers of different chemical concentrations was quantitatively studied. Although the preparation and loading of worms were manual in their configuration, the method offered advantages in setting up the assay and testing 0–300 mM of NaCl for attractive or repulsive responses. The authors showed that L3-stage worms exhibited a stronger response to low concentration NaCl as opposed to adult animals. Overall, the device can accelerate chemotaxis measurements in worms.

For detailed neurobiological studies of behavior, there is a need to observe changes in neuronal activities as worms interact with their environment. To this end, Hu et al. [61] fabricated a comb-shaped device that allowed in vivo monitoring of neurons using the GCaMP sensor in single immobilized worms as they were exposed to gases and odors. As a proof of principle, the authors examined two different types of neurons—URX that respond to oxygen (O_2_) and BAG that are sensitive to CO_2_ levels. In both cases, changes in Ca^2+^ signals were observed as expected. Additional sensory neurons were also successfully tested following exposure to odors such as 1-Octanol. Overall, the device is useful in studying neuronal responses in individual animals. In a different study Ca^2+^ sensor was used to identify magnetosensory neurons. For this a two-layer microdevice was fabricated containing a flow layer and valve layer. Worms were introduced via one end of the flow layer. For observation purposes they were fully immobilized by applying pressure in the valve layer. The animals were then exposed to a 65-Gauss (100× earth) rotating (2 Hz) magnetic field stimulus for 8 s and imaging was performed [62]. The result revealed that AFD neurons are specifically responsible for sensing the magnetic field. This mechanism may play a role in mediating the burrowing behavior of worms as they search for food in their natural environment.

Another stimulus that *C. elegans* is strongly sensitive to is the electric field. This was reported in 1978 by Sukul and Croll [63], who observed preferential movement of animals with an angle towards cathode. Prior to this discovery, electrotaxis phenomenon was described in other nematodes including *Panagrellus redivivus* and *Trichostrongylus retortaeformis* [64,65]. In spite of these early studies, no significant progress was made in this area until 2007 when Gabel et al. [18] reported amphid neurons and genes that mediate electrotaxis behavior using an open agar gel surface setup. Our group was the first to incorporate the electrotaxis assay in a microfluidic setup shown in Figure 6C. In the paper by Rezai et al. [9] we presented a straight 300 μm-wide microfluidic channel with end electrodes and used it to show that *C. elegans* responds to DC electric field and moves towards the cathode with a characteristic speed (roughly 350 μm/s in case of young adults). We also showed that a response to electric field is present starting at the L3 larval stage and that exposure to the field causes no harm to animals.

Microfluidic electrotaxis approach offers many advantages in investigating neuronal signaling and movement-related neuronal disorders (e.g., Parkinson’s disease-like phenotype [66]). It is the only non-invasive on-demand tool to induce instant directional movement with a defined speed. In subsequent studies we further characterized the electrotaxis phenomenon and showed that a shorter pulse of DC can also be effective in inducing robust movement in worms [67]. Interestingly, we found that worms are sensitive to the AC field as well but that the AC field produces a different response. Specifically, a symmetric square wave AC field of 1 Hz frequency was able to restrain animals effectively at one location [68], making it an attractive approach to implementing in a high throughput system where there is a need to localize and concentrate many worms. With the goal of accelerating microfluidic electrotaxis assays, we recently developed a semi-automated system [69]. At a throughput of 20 worms an hour, this system allows fast screening of mutants and drug-exposed worms with altered neuronal function. A custom LabVIEW program has been written to manipulate the steps of loading, capturing, flushing, releasing, electrotaxis screening, and channel cleaning. As a proof of principle, a mutant affecting dopamine signaling was successfully characterized.

### 3.4. Antimicrobial and Toxicological Studies

Other applications of microfluidic technology in *C. elegans* research include assessing the impact of toxic chemicals and harmful bacteria on living systems. As a whole organism model, the worm is frequently used as a bio-indicator in toxicological and environmental studies [70,71]. Typical exposure assays involve measuring growth rate, reproduction, movement, shape and size, neurodegeneration, and survival. The paper by Jung et al. [72] describes a device to measure the body volume of animals following exposure to toxic chemicals. Animals were immobilized in a tapered channel and size was measured by capacitance change using a pair of electrodes at the two sides of the tapered channel. As expected, the authors observed a reduction in size following exposure to cadmium. While useful, a limitation of this setup was that worms needed to be treated on standard Petri dish culture plates prior to doing measurements in the device. To address this, the authors later developed a new design that included on-chip exposure and collection chambers [73]. Animals were kept in one chamber and as they passed, one by one, through a narrow region containing electrodes, changes in capacitance were recorded and attributed to animals’ body sizes. This system accelerated chemical treatments and measurements of resulting changes in the body size.

The electrotaxis assay has also been extended to toxicological research. In one study, we examined the movement responses of worms in a microchannel device following exposure to toxins affecting dopaminergic (DAergic) neurons [74]. Animals with excessive neurodegeneration showed abnormal electrotaxis, demonstrating the utility of our assay in neurotoxicology studies. Ongoing cell biological experiments in our lab have identified components of the DA signaling pathway in mediating the electrotaxis behaviour [75].

Other researchers have also investigated the health of DAergic neurons of *C. elegans* in biosensor devices. Zhang et al. [76] used a microfluidic device consisting of eight culture chambers arrayed radially and connected in the center to a microfluidic chemical gradient generator similar in operation to the one shown in Figure 6B. The device could be used to measure survival and body stroke frequency of animals, as well as fluorescence intensities of neurons. Worms were exposed to varying concentrations of a heavy metal Manganese (Mg) (0 to 100 mM concentration range) and effects on movement and neurodegeneration were monitored. Exposure to natural antioxidants such as vitamin E, resveratrol, and quercetin were shown to rescue defects effectively.

Besides heavy metals and toxins, microfluidic has also been successfully implemented in experiments involving exposure to bacterial pathogens and screening of antimicrobial compounds. Yang et al. [77] developed a device consisting of 32 culture chambers. Each chamber accommodated approximately 15 worms. The animals were fed with cultures of *Staphylococcus aureus* bacteria for about 6 h and monitored subsequently for survival. As expected, the exposure caused the death of the entire population within three days, demonstrating the suitability of the device in such assays. Natural compounds were also screened for antimicrobial activities.

### 3.5. Learning and Memory Studies

Research has shown that *C. elegans* have the ability to learn from past experiences and can alter subsequent responses accordingly. For example, when animals were conditioned with NaCl in the absence of food they showed reduced chemotaxis response [78,79]. In 2005, Zhang et al. [80] reported for the first time a microdevice consisting of an eight-arm maze to test the learning preference of worms to bacterial strains. Results revealed that worms exposed to pathogenic bacteria learn to avoid them within a few hours by recognizing associated odors while increasing their attraction to odors from familiar nonpathogenic bacteria. This learning process is mediated by serotonin in a specific chemosensory neuron, the ADF. In another study Qin and Wheeler [81] fabricated new devices to test olfactory learning of worms. These devices, consisting of T-shaped and U-shaped patterns, were made to examine the behavior of worms as they explore the environment and choose to go in a particular direction. It was found that worms use olfactory stimuli to make biased movement. This behavior was also dependent on the dopamine neurotransmitter.

### 3.6. Reproductive Aging and Lifespan Studies

As discussed above, microfluidic technology is well suited for long-term culturing and observations of *C. elegans*. Given that these animals typically produce offspring during the first 3–4 days of their life and live for roughly three weeks, microfluidics holds significant potential to accelerate reproductive aging and lifespan-related studies.

The device by Hulme et al. [23], discussed above (Figure 2A), is not only useful in culturing worms but can also allow monitoring of age-associated changes. Two parameters, body size and locomotion, were monitored and found to be in agreement with the results derived from standard plate-based studies. Another study by Xian et al. [82] described an integrated multilayer PDMS device platform, termed WormFarm, for aging studies. This setup consisted of eight chambers (each 3 × 10 mm^2^) that were separated to avoid cross-contamination. Each chamber could hold approximately 40 worms. Chambers were flushed periodically to remove progeny and supply fresh bacteria to feed adults. Worms were counted using a custom algorithm. Additionally, GFP fluorescence in aging animals could also be quantified as average signal intensity per pixel. The device allowed rapid quantification of changes in lifespan caused by environmental and genetic manipulations.

**Figure 7:**
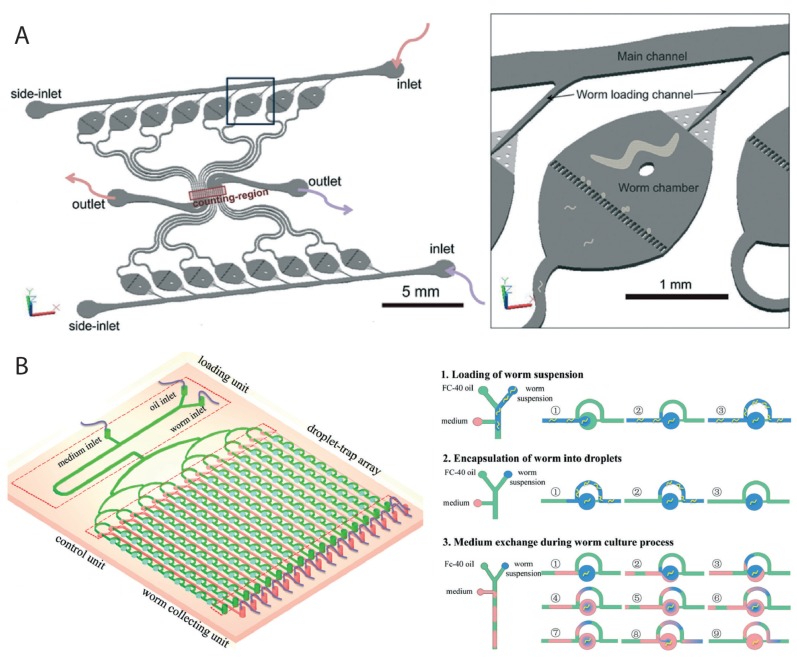
Microfluidic devices to investigate *C. elegans* (**A**) egg-laying (Li et al. [83]) and (**B**) development (Wen et al. [30]), by isolating worms inside microchambers with renewable chemical environment. Reproduced with permission from the Royal Society of Chemistry. The left-hand diagram in panel (**A**) shows eight chambers on each side (one of which is enlarged on the right). Inlets are used to load worms and outlets for bacterial flow. The middle counting region (2 mm × 2 mm), indicated by the red rectangle, is monitored by camera. Panel (**B**) shows the droplet chip. Schematics of worm encapsulation and substrate exchange in each droplet are shown in three steps on the right side. The amount of substrate exchange is indicated by the color change. Refer to respective references for more details.

Two other devices have also been reported recently. One of these, described in Li et al. [83], was used to automate the counting of progeny from several worms simultaneously, which is normally a manual procedure that is tedious to perform. The device (Figure 7A) consists of 16 microchambers, each housing a single *C. elegans* hermaphrodite (wild type or *daf-*2 insulin receptor mutant). The chambers were fed with bacteria and simultaneously washed to direct the laid eggs through a filter to an automated and real-time progeny counting module in the center of the device. The system re-confirmed previous published findings, specifically the reproductive period of *daf-*2 mutants being longer than that of wild-type animals. In the future this promises to accelerate the study of reproduction and reproductive aging in a real-time manner. In the other paper, by Wen et al. [30], the authors took a different approach that involved formation of fluorocarbon oil (FC-40)-based microdroplets to monitor individual animals (Figure 7B). Worms were encapsulated into droplets (one per droplet) at the L1 stage using a clever design and growth was monitored over time. Fast exchange of media and substance were possible several times a day to allow worms to live normally till adulthood. The device can be useful in studying changes such as body shape, length, and developmental delays in large synchronous populations following certain manipulations. For instance, the authors showed that the worms overexpressing *hif-*1 (hypoxia-inducible factor) had a short body length and a slow growth rate, which indicates the possibility of HIF-1 being involved in developmental processes.

Chuang et al. [84] recently reported an interesting application of an electrotaxis-based microfluidic flow chamber to study the effect of exercise on age-related degenerative changes. Their device, termed a “worm treadmill,” involved inducing directed movement in worms using a DC electric field stimulus. Worms were allowed to swim for 10 min without interruption once every day for a total of eight days, and the effects on various age-related processes were subsequently monitored. The results showed that exercise improved the overall health of animals, as judged by a number of factors such as increased size and density of mitochondria, reduced oxidative stress, and prolonged life span.

In summary, microchamber environments hold a significant promise for a range of lifespan-related and developmental assays. Advantages offered include phenotypic studies at the single animal level, micro-compartments to manage the number of animals for a given study, effective control of microenvironments, being able to separate offspring from mothers, and the ability to monitor behavioral and neuronal activities over an extended period of time.

## 4. Microfluidic Devices to Perform Fast, Automated, and High-Throughput Screening

The small size and short life cycle of *C. elegans* make it highly suitable for high-throughput screening to investigate mechanisms of diseases and identify candidate drugs for further validations in other model organisms. As the *C. elegans* genome contains more than half of human disease-related gene orthologs including genes implicated in cancers, diabetes, and neurodegenerative diseases [85], microfluidic tools hold great potential to automate *C. elegans* screening, thereby accelerating the development of effective treatments for human diseases. Via integration of automation micro-components (e.g., micropumps and microvalves) and a variety of nontoxic worm-compatible materials (e.g., PDMS elastomer, glass, and hydro- and responsive gels), several high-throughput devices have been developed for phenotypic screening, microsurgery, and drug exposure of *C. elegans*.

### 4.1. Rapid and High-Throughput Screening

Microfluidic devices are appropriate tools for high-throughput neuro-behavioral screening of worms and subsequent sorting based on size, mutation, or response to stimuli. Neuronal screening has been achieved by immobilizing the worms in microchannels, followed by the fluorescent or electrophysiological recording of neurons’ static or dynamic responses before sorting takes place. Behavioral screening has also been achieved by investigating the crawling, swimming, and egg laying responses of an individual or a group of worms to external stimuli, applied controllably inside fluidic microenvironments. Fast and viable delivery of animals to desired interrogation locations on a chip is a critical unit operation in these assays.

A microvalve-controlled “Population Delivery Chip” was developed to rapidly deliver 16 different worm populations, each under 4.7 s, to favorable locations on a microchip to accelerate screening [86]. This technique can be used in assays where a specific population of synchronized worms is needed for downstream high-throughput investigations. In this regard, Rohde et al. [21] developed a microfluidic system for high-speed sorting and subcellular phenotypic screening of worms. Pre-synchronized worms were captured in a microchannel by immobilization via side suction channels and imaged at high resolution. To expand the utility of their platform, the authors developed an interface chip consisting of 96 aspirator tips to connect the main screening device to 96-well plates. This setup could be used to carry out chemical and RNA interference (RNAi) screening, although no such screening was actually demonstrated in the paper. Chung et al. [20] also developed a similar device but with several differences. In their setup, worms could be rapidly immobilized in a channel for high-resolution imaging of neuronal processes by lowering the temperature of the channel using a Peltier cooler. The entire setup was automated and could operate at a speed of several hundred worms per hour. A variation of this device (without the cooling unit) was later combined with a computer-assisted setup for automated and faster genetic screening of mutants [87]. Another paper by Crane et al. [88] also reported development of an automated platform for fast screening of neuronal phenotypes. The setup was designed to load worms, image neurons using GFP markers, process fluorescence data using computer software, and perform sorting based on the analyzed data. A Peltier cooler approach (similar to [20]) was used to transiently immobilize animals for ~10 s in order to capture and process the images. Animals having desired phenotypes were collected in the end. This entire operation was performed without a camera and microscope. Moreover, very little human intervention was required. A throughput of more than 220 worms per hour was demonstrated, which is suitable for large-scale mutant, RNAi, and drug screening for neuronal function.

In certain screening it is desirable to orient worms in a specific manner, which cannot be achieved in the abovementioned devices. Cáceres et al. [89] have demonstrated that orientation along the dorsal–ventral body axis can play an important role in the inspection of morphological features with specific dorsal–ventral alignments. Their device was capable of orienting worms laterally in a U-shaped microchannel with high frequency such that neuronal processes could be screened at the rate of roughly 500 worms an hour. Several new mutants affecting motor neurons were recovered in this study.

The above devices rely on physical or thermal contact with animals in order to immobilize them. Recently, Yan et al. [90] developed an approach in which individual worms with different levels of GFP expression were encapsulated in oil droplets and analyzed fluorescently in an automated setup. The system provided 100% accuracy and an analysis speed of 0.5 s per worm, although resolution was poor.

A limitation of the screening systems discussed so far in this section is that live imaging of transient cellular processes in active animals cannot be performed. Chokshi et al. [91] reported an automated high-speed platform to image neuronal activity using transient calcium imaging in live worms exposed to different odors. The platform could screen tens to hundreds of worms per hour. As a test case, calcium responses were measured in worms of different ages exposed to hyperosmotic stimulus (1 M glycerol). The results revealed that the activity of a specific chemosensory neuron ASH was significantly diminished in older animals. Similar screening may be performed for other odors and neurons.

**Figure 8:**
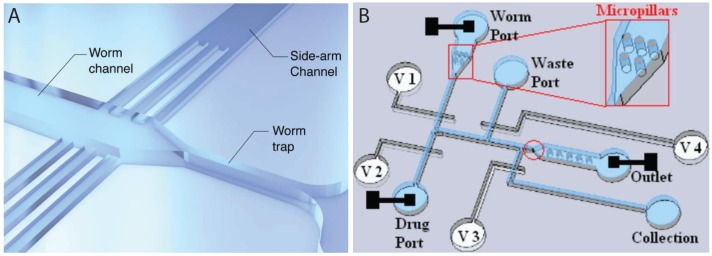
Microfluidic devices for neuromuscular electrophysiological studies on *C. elegans* (Lockery et al. and Hu et al. [92,94]). Panel (**A**) reproduced with permission from the Royal Society of Chemistry. The EPG recording device (panel (**A**)) contains a worm channel and a funnel-shaped trap region. Fluid flows through the side-arm channel. Panel (**B**) shows the neurochip. Blue indicates the layer containing the microfluidic region (for worms and chemicals) and white shows the pneumatic control layer. The red circle contains the trapped worm’s head. The red square contains micropillars to correctly orient the worm. V1–4 are valves and the solid black squares are microelectrodes. Refer to respective references for more details.

While fluorescent microscopy based on GFP or GCaMP sensors has led to a number of very useful microfluidic devices for high throughput studies, two recent papers have demonstrated the power of PDMS-based microfluidics in non-invasive electrophysiological readout of neuromuscular activities. These devices could provide direct information about the target of drug action, and are amenable to parallelization. Lockery et al. [92] developed a device (Figure 8A) to record electrophysiological activities of pharynx (termed electropharyngeogram or EPG), a neuromuscular organ involved in feeding. The device consisted of a funnel-shaped channel to clamp the pharynx of a single worm, electrodes at the anterior and posterior sides to record the EPG signal, and side channels for perfusion of chemicals into the device to expose the worm. Although the system was not fully automated, as it required placing worms manually into the inlet port, it could, however, perform recordings of eight worms simultaneously, which is significant compared to existing manual methods [93]. Although no screening was demonstrated, the setup offers a rapid and sensitive way to identify chemicals, e.g., anthelmintics, which may affect the physiology of worms. Another similar device (Figure 8B) for neurobiological studies in *C. elegans*, including neurotoxicology and drug screening, was developed by Hu et al. [94]. Worms were exposed to chemicals and EPG responses were monitored along with neuronal activities using fluorescent probes. Worms could also be recovered, which makes it possible for phenotypic screening. A throughput of 12 worms per hour demonstrated that it is significantly faster than conventional EPG protocols.

### 4.2. Drug Delivery and Behavioral Screening

Developmental and physiological changes such as crawling, swimming, and egg laying are widely used readouts in *C. elegans* assays. Screening based on such features has been successfully demonstrated in microfluidic environments.

Chung et al. [95] developed a device that contains parallel arrays of worm chambers to facilitate behavior-based chemical screening at single-animal resolution. A total of 48 chambers, each 1.5 mm diameter, were designed in an 8 (column) × 6 (row) single layer format. A serpentine channel of 500 μm width was used to deliver worms and media to each chamber. All chambers are visible in the field of view, making it possible to monitor individual worm responses following exposure to chemicals. Using this setup, researchers succeeded in measuring changes in behavioral activities, such as body bending and mating, following exposure to drugs and sex pheromones. The embryo-culturing platform of Cornaglia et al. [39] discussed before (Section 2.3) can also be used for fast and automated screening of biological processes and drugs. The setup contains two separate regions for embryo and worm cultures that are connected via a microchannel. The animals could be maintained in the device for extended periods of time and periodically imaged as desired. Up to 20 single embryos could be arrayed automatically upon delivery and monitored simultaneously, making it possible to monitor gene expression and developmental events. It is possible to visualize the entire embryogenesis using this device. The platform was successfully used to study mitochondrial biogenesis and mitochondrial unfolded protein response (UPR) processes.

In addition to egg-laying, locomotion behavior has also been investigated in a medium-to-high throughput manner using microfluidic devices. Three reports have successfully implemented electrotaxis behavior to achieve these goals [69,74,96]. Carr et al. [96] developed a microfluidic device to perform drug screening using electrotactic motion as a readout. The chip contained a reservoir to expose worms to defined chemicals and a microchannel with electrodes across it to perform electrotaxis to investigate their movement responses. Levamisole, an anthelmintic drug, was used as a test case to demonstrate the successful use of the device for *C. elegans* as well as a parasitic nematode *Oesophagotomum dentatum*. In the paper by Salam et al. [74], our group characterized the electrotaxis defects of neurotoxin-treated worms. The neurotoxins caused damage to dopaminergic (DAergic) neurons, resulting in movement defect and serving as a model for the study of Parkinson’s disease. To accelerate screening of electrotaxis phenotypes, we recently developed an advanced version of this device [69] by automating the process of single worm loading, stabilizing the fluid flow, exposing the worm to electrical signals, and recording and analyzing the electrotactic response.

Overall, these studies show that behavioral and movement-based microfluidic screening approaches can be used as platform technologies for automated drug screening applications in neurodegenerative and muscular diseases. An interesting direction in the future may involve simultaneous screening of neuronal and behavioural activities following exposure to chemicals. This will enable correlation of sensory and motor outputs in order to develop more specific treatments.

### 4.3. Microsurgery, Regeneration, and Drug Screening

**Figure 9:**
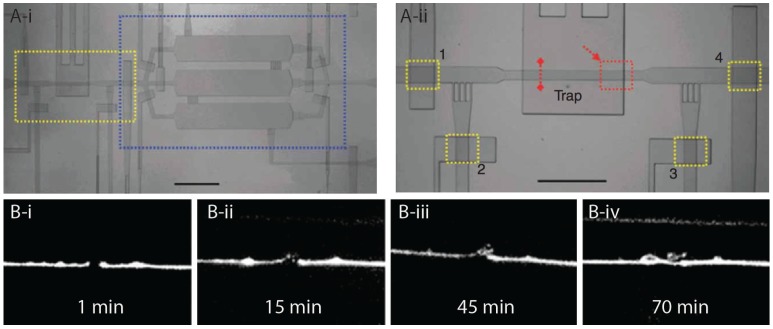
A microfluidic device for (**A**) laser nanoaxotomy of the ALM neuron in *C. elegans* and (**B**) investigation of time-lapse nerve regeneration (Guo et al. [97]). Reproduced with permission from Adapted by permission from Macmillan Publishers Ltd. Nature Methods. On the left (**A-i**) the trap system (**yellow** rectangle) and the three recovery chambers (**blue** rectangle) are indicated. The right panel (**A-ii**) shows a magnified view of the trapping system. The small yellow dotted rectangles show four valves to control worms. Panels (**B-i to B-iv**) show axonal recovery. Branching is visible several minutes after axotomy. By 70 min the nerve has regrown and appears to be reconnected. Refer to the references for more details.

Nanoaxotomy of neurons and whole cell ablations are highly delicate processes, especially when it comes to performing such operations on the micron-size *C. elegans*. As discussed above, worm manipulation and immobilization can be achieved in an automated, high-throughput, and very precise manner using microfluidics. These assets have made microfluidic devices very effective and useful in laser nanoaxotomy and nerve regeneration studies of *C. elegans*. Guo et al. [97] and Zeng et al. [98] have developed nanoaxotomy chips for fast surgical operations of nerves, in order to study nerve regeneration and perform high-throughput screening. Both setups were designed to trap worms by applying pressure on a flexible PDMS membrane (Figure 9A). This allowed fast operation and recovery of worms after nanosurgery. GFP fluorescence tags were used to locate neurons to perform surgery and monitor the regeneration processes (Figure 9B). A throughput of 60 worms per hour (from time of loading the worm to performing surgery) has been reported [97]. It was shown that axonal regrowth occurs faster in the distal fragment in the absence of anesthetics. Later on, Chung and Lu [99] used their cooling immobilization technique to develop a microfluidic device to rapidly paralyze L1 stage worms in two parallel traps and ablate neuronal cell bodies sequentially. The two-trap system greatly increases the speed of operation. While one trap loads a worm, the other is used to perform the surgery. This is accomplished by an automated setup that moves the stage to align the desired worm with the laser beam. The authors reported a throughput of 110 worms per hour, which was enough to obtain a population of animals for behavioural assays. Samara et al. [100] combined microfluidics and femtosecond laser microsurgery to perform semi-automated loading and single-axon ablation in *C. elegans* at a rate of approximately 180 worms per hour. Their immobilization approach involved first orienting the worm using an array of suction channels and then applying pressure on the top PDMS layer membrane to fully constrain the animal motion. The post-ablated worms were exposed to a small library of chemicals to screen for candidates with regenerative effects. A total of 10 drugs were identified that significantly enhanced the neurite regeneration.

Another device to perform high throughput laser axotomy was developed by Gokce et al. [101]. The device contains a loading chamber for housing up to 250 synchronized stage worms. The chamber is connected to a staging area that allows trapping of single worms for axotomy. The animals were immobilized by a deflecting membrane technique that physically confines them in a small region. The entire process of animal loading, staging, neuron identification, axon ablation, and unloading was fully automated with the help of computer-controlled microfluidic components and image processing tools. An average throughput of about 210 worms per hour was reported for ablation of 300 nm-wide axons at sub-micron resolution.

All in all, researchers have successfully developed automated microfluidic systems for rapid surgery of axons and ablation of neuronal cell bodies in *C. elegans* and post-operated manipulations of such animals. The existing technologies are summarized in Table 2 for an easy comparison. The reported throughputs are an order of magnitude faster than the conventional manual methods. These developments are expected to facilitate the study of nerve regeneration and perform drug screening.

## 5. Concluding Remarks

Microfluidics offers powerful tools to accelerate fundamental and drug discovery research in *C. elegans*. This review provides a comprehensive summary of major devices and platforms that are enabling applications ranging from the routine handling of animals to fast, automated, and high-throughput screenings. We have primarily focused on the technological aspects of the progress to align with the audience of this journal. The description of experimental data is largely meant to support the usefulness of approaches.

Despite the excellent progress in the above fields, there are a few hurdles for greater acceptance of the technology by mainstream *C. elegans* labs. At present, the technical know-how about microfluidic systems and the infrastructure needed to produce devices is restricted mainly to labs with significant engineering expertise. Other hurdles include the high cost of fabrication, the need for customized hardware and software to process data, and the manual work required to operate many of the devices. Overcoming these barriers will require training the next generation of *C. elegans* scientists to be comfortable with fabricating and operating custom microdevices.

Although virtually all areas of *C. elegans* research have been miniaturized, there are a few where growth has been rapid and impressive. These include in vivo analysis of neuronal function, longitudinal studies of gene expression, and age-related processes. Among the various approaches, a genetically encoded GCaMP Ca^2+^ sensor in combination with pressure-based and temperature-sensitive gel-based immobilization offers powerful means to accelerate in vivo studies of cellular processes at high resolution. The electrotaxis response is also a promising tool to manipulate worms in different ways including sorting, transport, and exercise treatments. We expect to see a lot more progress in these directions, including improved devices for characterization of disease models and gene target identifications that are relevant to humans.

Drug discovery is one frontier where microfluidics holds significant potential and *C. elegans* screening can help in the identification of candidate targets in a rapid and cost-effective manner. To this end, several devices have been developed (e.g., [20,21,91]) that are capable of performing high-throughput assays. The system by Cornaglia et al. [39] is interesting as it allows monitoring of the whole of embryogenesis following chemical exposures and other manipulations. Axonal regeneration and neurodegenerative disease model-based screenings are also expected to yield exciting results in the near future due to the ease of visualizing neuronal morphologies in live animals. We anticipate that high-throughput platforms such as Samara et al. [100], Gokce et al. [101], and Cornaglia et al. [31] will enable new screening to identify drug candidates as well as genetic targets for further validations in higher eukaryotes. Overall, *C. elegans* microfluidics research holds significant promise to advance our understanding of human diseases and to help develop potential treatments.

## Figures and Tables

**Table 1:** Overview of microfluidic devices to enable sorting of *C. elegans*.

Device	Method	Throughput	Sorting Capabilities	References
PDMS single channel device	Electrotaxis	78 worms per minute	Specific developmental stages and adults; mutants from wild type	Rezai et al., 2010 and 2012 [9,10]
Agarose gel box containing a single long channel	Electrotaxis	Unknown	Adults of different age; mutants from wild type	Maniere et al., 2011 [11]
PDMS device containing interconnected mazes	Flow filtration	200–300 worms per minute	Larvae from adults	Solvas et al., 2011 [12]
PDMS multichannel device	Electrotaxis	4 worms per minute	All stages including adults	Han et al., 2012 [13]
PDMS micro-pillar device	Flow filtration	130–180 worms per minute	All stages including adults	Ai et al., 2014 [14]
PDMS device containing optical fiber and laminar flow switch	Fluorescence filtration	12 worms per minute	Fluorescent animals	Yan et al., 2014 [15]
PDMS-Agarose fan-shaped device	Electrotaxis	56 worms per minute	L2–L4 larvae and adult; size-based separation	Wang et al., 2015 [16]
PDMS device containing adjustable filter	Pressure-based filtration	200 worms per minute	Certain developmental stages	Dong et al., 2016 [17]

**Table 2:** Overview of microfluidic devices to perform microsurgery in *C. elegans*.

Immobilization Method	Throughput	Operation Performed	References
Pressure-based	60 worm per hour	Neuroaxotomy and nerve regeneration	Guo et al. [97]
Pressure-based	One worm every few seconds	Neuroaxotomy	Zeng et al. [98]
Cooling	120 worms per hour	Cell ablation	Chung and Lu [99]
Suction- and pressure-based	180 worms per hour	Neuroaxotomy and nerve regeneration	Samara et al. [100]
Pressure-based	210 worms per hour	Neuroaxotomy	Gokce et al. [101]
